# Miscibility and Solubility of Caffeine and Theophylline in Hydroxypropyl Methylcellulose

**DOI:** 10.3390/pharmaceutics13111836

**Published:** 2021-11-02

**Authors:** Edyta Leyk, Marek Wesolowski

**Affiliations:** Department of Analytical Chemistry, Faculty of Pharmacy, Medical University of Gdansk, Gen. J. Hallera 107, 80-416 Gdansk, Poland; edyta.leyk@gumed.edu.pl

**Keywords:** caffeine, theophylline, HPMC, miscibility curves, solubility curves, DSC, HSM, FTIT, Raman spectroscopy, principal component analysis

## Abstract

As amorphization may improve the solubility and bioavailability of a drug substance, the aim of this work was to assess to what extent the crystallinity of caffeine (CAF) and theophylline (TF) can be reduced by homogenization with a polymeric excipient. To realize this purpose, the physical mixtures of both methylxanthines with hydroxypropyl methylcellulose (HPMC) were examined using differential scanning calorimetry (DSC), hot-stage microscopy (HSM), Fourier-transform infrared (FTIR) and Raman spectroscopy. Moreover, phase diagrams for the physical mixtures were calculated using theoretical data. Results of DSC experiments suggested that both CAF and TF underwent amorphization, which indicated proportional loss of crystallinity for methylxanthines in the mixtures with HPMC. Additionally, HSM revealed that no other crystalline or amorphous phases were created other than those observed for CAF and TF. FTIR and Raman spectra displayed all the bands characteristic for methylxanthines in mixtures with HPMC, thereby excluding changes in their chemical structures. However, changes to the intensity of the bands created by hydrogen bonds imply the formation of hydrogen bonding in the carbonyl group of methylxanthines and the methyl polymer group. This is consistent with data obtained using principal component analysis. The findings of these studies revealed the quantities of methylxanthines which may be dissolved in the polymer at a given temperature and the composition at which methylxanthines and polymer are sufficiently miscible to form a solid solution.

## 1. Introduction

The use of polymorphous or amorphous forms, organic salts, co-crystals or solid dispersions usually improves the bioavailability of poorly water-soluble active ingredients (drug substances) [1]. Amorphization is advantageous because the creation of an amorphous form leads to the creation of extremely small particles. [2,3,4]. The solubility of this form is usually profitable because no energy is needed to break the crystal lattice. Solid dispersions (SDs) of drug substances with polymeric excipients are also an attractive way to improve the solubility and bioavailability of active substances [2,3]. The drug substance is dispersed at a molecular level in a solid, usually polymeric matrix [3,4]. As shown in Table 1, reports regarding pharmacokinetics of drug substances administered orally to animals in the form of SDs with polymers revealed that the area under the curve (*AUC*) and maximum blood concentration (*C_max_*) are, respectively, 1.6–3.2 and 1.5–34.9 times higher for SDs than for crystalline drug substances [5,6,7,8,9,10,11]. Moreover, the time required to reach maximum blood concentration (*T_max_*) is reduced by 1.2–3.5 times. It may be valuable therefore to explore whether polymeric excipients affect the crystallinity of methylxanthines. Understanding the nature of interactions in the solid state between ingredients of SDs constitutes a contribution to the development of pharmaceutical technology and one which may create new, more advantageous formulations.

Solid dispersions are usually obtained by melting a drug substance mixture with polymer or by evaporating a solvent from drug substance solution with polymer. The hydrogen bonds often formed between the active substance and the polymer stabilize the system and inhibit the crystallization process [2,12,13]. The possibility to obtain SDs can be predicted from the physicochemical properties of ingredients such as crystallinity and type of interactions between components. In the case of mixtures of active substance with polymeric excipient, a phase behavior of ingredients can be extremely complicated because the active substance can be in a crystalline, partially amorphous or amorphous form [14]. Thus, a phase diagram for mixtures of crystalline active substance with amorphous excipient reflects the solubility of the crystalline drug substance in amorphous excipient and the miscibility of that part of the drug substance which underwent amorphization in amorphous excipient [14,15,16,17]. If the active substance is in completely amorphous form, a glass transition curve may also appear. The construction of phase diagrams is a theoretical assumption based on mathematical calculations. In the crystalline drug substance–amorphous polymer solid systems, miscibility describes a single phase of homogenous system, in which active substance and polymeric excipient are intimately mixed at a molecular level [14]. Both ingredients are in an amorphous form. In other words, miscibility shows a tendency of the fused or amorphous drug substance to disperse in polymeric excipient. The miscibility curve defines the upper limit of drug substance loading which does not lead to spontaneous phase separation followed by drug substance crystallization. On the other hand, solubility shows an ability of the polymeric excipient to dissolve crystalline drug substance. In this case polymer serve as a solvent [14]. The solubility curve illustrates the maximum loading of drug substance that creates a thermodynamically stable solid solution. The amount of drug substance that can be dissolved in the polymer depends on the physicochemical properties of the ingredients, their proportion and the temperature of the mixture. So, partial drug substance dissolution results in a partial loss of crystallinity. Assuming that the amorphous polymer is a solvent, the solubility curve of crystalline drug substance is a visual representation of the quantity of the crystalline active substance that can be dissolved in the polymer.

**Figure 1:**
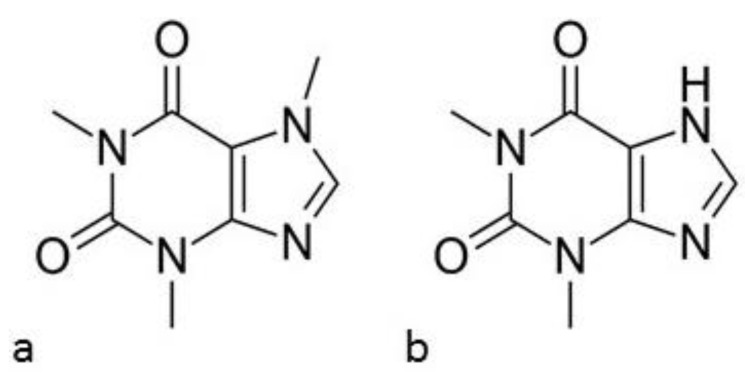
Chemical structures of (**a**) caffeine and (**b**) theophylline.

The aim of this work was to predict to what extent the crystallinity of methylxanthines can be reduced by homogenization with polymers used in pharmaceutical solid drug dosage technology. For this purpose, phase diagrams were constructed for physical mixtures (PMs) of caffeine (CAF) and theophylline (TF) in the solid state with hydroxypropyl methylcellulose (HPMC, hypromellose). Caffeine is an analeptic agent that is used in migraine therapy [18] and theophylline is applied to prevent asthma attacks and to treat bronchitis and pneumonia [19]. Chemical formulas of CAF (1,3,7-trimethylxanthine; 1,3,7-trimethylpurine-2,6-dione) and TF (1,3-dimethylxanthine; 1,3-dimethyl-7H-purine-2,6-dione) are shown in Figure 1. Both drug substances are weakly acids with acidity constant (*pK_a_*) at 14.0 and 8.8 and lipophilicity (octanol-water partition coefficient, *LogP*) at −0.1 and −0.2 for caffeine and theophylline, respectively [20]. Both methylxanthines were chosen for the study because they are low water-soluble substances, 1:60 and 1:180 for CAF and TF, respectively [20,21]. Amorphization can lead to improve their pharmacokinetic properties, that is *AUC*, *C_max_* and *T_max_*. In addition to amorphization, various means have also been sought to improve solubility of methylxanthines. For example, the formation of SDs or PMs with polymeric excipients such as methylcellulose and chitosan leads to a reduction in TF crystallinity [22,23]. This phenomenon may be related to the formation of hydrogen bonds between the amino group of chitosan and the carbonyl group of theophylline [23]. To confirm the reduction of crystallinity of CAF and TF in contact with HPMC, differential scanning calorimetry (DSC) and hot-stage microscopy (HSM) were employed. Fourier-transform infrared (FTIR) and Raman spectroscopies were also applied to assess the precise nature of the interactions between methylxanthines and polymeric excipient.

## 2. Materials and Methods

### 2.1. Drug Substances and Polymer

Caffeine (CAF), theophylline (TF) and hydroxypropyl methylcellulose (HPMC, 28–30% and 7–12% of methoxy- and hydroxypropoxy-groups, respectively), were obtained from Sigma-Aldrich (Steinheim, Germany). All substances were ≥99% grade and used as received.

Binary physical mixtures (PMs) in the solid state of CAF or TF with polymeric excipient containing 10%, 30%, 50%, 70% and 90% of HPMC were prepared by thorough homogenization of ingredients in a porcelain mortar using a plastic spatula (20 min). All substances were weighed using XA 105 Mettler Toledo Dual Range instrument (Schwerzenbach, Switzerland).

### 2.2. Calculations for Phase Diagrams

The miscibility curve was developed based on the assumption that a mixture with a well-defined proportion of ingredients is thermodynamically stable at a given temperature. This curve was predicted on the basis of the Florry–Huggins theory and the Gibbs free energy, according to the calculation method presented in the papers [14,15,21,24,25]. In order to develop the solubility curve, the quantity of crystalline substance that can be dissolved in a polymer at a given temperature was determined. The melting point, the heat of fusion and the Hansen solubility parameters were used for the calculations, according to the approach presented elsewhere [15,26,27]. Literature data on the solubility parameters indicating intermolecular interactions were also used in the calculations [28,29,30]. These parameters include the dispersion forces and the polar and hydrogen interactions [21,24,25]. Furthermore, the density, molar masses and lattice sites of crystalline methylxanthines are also taken from the literature [28,29,30,31,32]. The values used in the calculations are listed in Table 2.

### 2.3. Differential Scanning Calorimetric Study

A DSC study was performed by a Mettler Toledo heat-flux DSC 822e device (Schwerzenbach, Switzerland), equipped with Dewar vessel and cooled with liquid nitrogen and controlled by STARe 15 software. About 4 mg (±0.01 mg) of sample was weighed into an aluminum pan with a pin in the lid. An empty pan was used as a reference. Measurements in triplicate were performed under nitrogen (purity 99.9997%, Air Products, Warsaw, Poland) at a flux rate of 70 mL/min. The samples were heated in the range of 25–300 °C at a heating rate of 10 °C/min.

Indium (In, purity 99.999%) and zinc (Zn, purity 99.998%), both standards from Mettler Toledo (Schwerzenbach, Switzerland), were used to calibrate a DSC cell. Reference values for the heat flow (Δ*H*) and the onset temperature (*T_on_*) were 28.45 J/g and 156.6 °C (In) and 107.5 J/g and 419.6 °C (Zn), while the values measured were 28.46 ± 0.32 J/g and 156.65 ± 0.15 °C (In), 107.67 ± 2.17 J/g and 419.64 ± 0.16 °C (Zn).

### 2.4. Hot-Stage Microscopic Study

A HSM test was carried out in the range of 25–300 °C at a heating rate of 10 °C/min using a BX41 Olympus polarizing microscope (Shinjuku, Japan). A color video digital camera SC30 with Olympus CellA software was used to record imagines during temperature scans. About 3 mg of sample was placed between two thin (0.13–0.17 mm) glass slides and put on a hot stage (Semic, Bioelektronika, Krakow, Poland). The heating block was equipped with an SR90 temperature regulator (Shimaden, Tokyo, Japan) and controlled by Heating Desk Shimaden software.

Temperature calibration in the HSM measurements was performed using glutaric acid (m.p. = 98 °C), indomethacin (m.p. = 162 °C), succinic acid (m.p. = 188 °C) and caffeine (m.p. = 236 °C). All substances were purchased from Sigma Aldrich (Steinheim, Germany). The strong relationship between the melting points acquired from HSM and those determined by DSC is described by the equation y = 0.804x + 3.695 (correlation coefficient r = 0.9990).

### 2.5. Spectroscopic Study

Infrared spectra were recorded with a Thermo Fischer Scientific Nicolet 380 FTIR spectrometer (Madison, WI, USA), controlled by OMNIC software and equipped with a deuterated triglycine sulfate (DTGS) detector with KBr window. A 1 mg sample was gently mixed in an agate mortar with 100 mg spectroscopy-grade KBr (Merck, Darmstadt, Germany) and then pressed into pellet form with a hydraulic press (Specac, Orpington, UK). The FTIR spectra were measured in triplicate in the spectral range of 4000–400 cm^–1^ with resolution of 2 cm^–1^ (16 scans). The background spectrum was recorded before each measurement was taken.

The Raman spectra were registered on a Thermo Fisher Scientific DXR SmartRaman spectrometer (Madison, WI, USA), with a Raleigh filter, charge-coupled detector (CCD) and OMNIC software. Measurements in triplicate were performed in the spectral range of 3413–99 cm^–1^ with a spectral resolution of 2 cm^–1^. The spectrometer was equipped with 15-mW DXR 780 nm laser (aperture of 25 µm) and samples were exposed to laser light for a period of 1 s.

### 2.6. Principal Components Calculations

A principal components analysis (PCA) of the data acquired from FTIR spectra was performed using Statistica 13.3 software (TIBCO Software Inc., Palo Alto, CA, USA). For PCA calculations, the absorbance values were collected every 2 cm^–1^ in the spectral ranges of 670–800 cm^–1^ and 1500–1800 cm^–1^. Prior to calculations, FTIR data were pre-processed using a standard normal variate algorithm (SNV) [33]. Two matrices were prepared for each methylxanthine (CAF and TF), i.e., four matrices in total. The first consisted of the FTIR data collected for both methylxanthine and HPMC over the course of the following four days, while the second was formed by the FTIR data collected for binary physical mixtures of methylxanthine containing 10%, 30%, 50%, 70% and 90% of HPMC. The results of PCA calculations were illustrated by 2D score scatter plots of the first two principal components (PC1 and PC2) and by the corresponding PC1 and PC2 loading profiles. Together the PC1 and PC2 explained more than 86% and almost 100% of the total variability for the first and the second matrix, respectively.

## 3. Results

### 3.1. Caffeine Mixtures with HPMC

**Figure 2:**
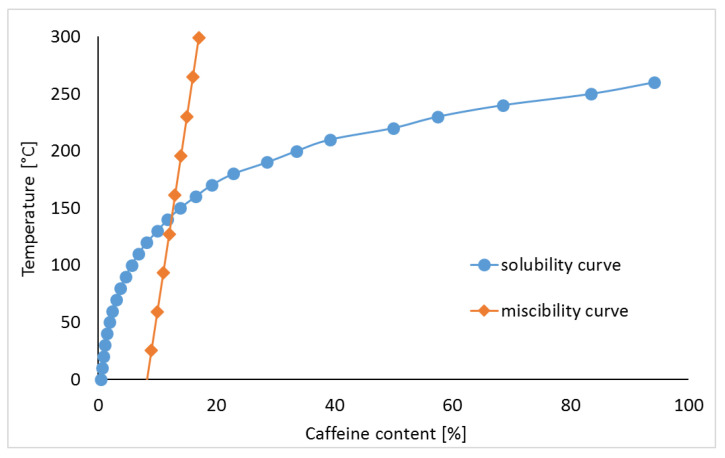
Phase diagram for caffeine binary mixtures with HPMC.

The phase diagram for mixtures of crystalline CAF with amorphous HPMC, including miscibility and solubility curves, is shown in Figure 2. The miscibility curve was predicted on the assumption that a mixture with a well-defined proportion of components, is thermodynamically stable at a given temperature [14,15,21,24,25]. This curve can be determined using the Florry–Huggins theory and the Gibbs free energy ($∆G_{mix}$) expressed by Equation (1):

(1) $$∆G_{mix}=RT\left[\phi_{API}\ln\phi_{API}+\frac{1-\phi_{API}}{n}\ln\left(1-\phi_{API}\right)+\chi \phi_{API}\left(1-\phi_{API}\right)\right],$$

where *n* is the number of drug substance lattice sites, defined as the volume of drug substance molecule occupied by a polymer chain and *χ* is the drug substance–polymer interaction parameter.

To develop the miscibility curve, temperatures ranging from 0 °C to the melting point of CAF were used. As shown in Table 3, the findings obtained reveal the temperatures at which the mixture containing predefined component ratios exhibits total miscibility.

The amount of active substance that can be dissolved in polymer was calculated using the mole fraction of drug substance that can be dissolved in the polymer ($x_{API}$) [15,26]:

(2) $$\ln x_{API}=\frac{∆H_{f}}{RT_{m}}\left(1-\frac{T_{m}}{T}\right)-\ln\gamma_{API},$$

where $\gamma_{API}$ is the activity coefficient, $∆H_{f}$ the heat of fusion of drug substance, $T_{m}$ the melting point of drug substance, R the gas constant and T the temperature of ingredients in equilibrium.

Hansen solubility parameters (δ) were used to calculate the drug substance coefficient ($\gamma_{API}$). Solubility parameters indicate intermolecular interactions and are differentiated into dispersion forces ($\delta_{d}$), polar interactions $\delta_{p}$ and hydrogen bonding ($\delta_{h}$):

(3) $$\ln\gamma_{API}=\frac{V_{API}}{RT}\left\{{\left(\delta_{d}^{API}-{\bar{\delta }}_{d}\right)}^{2}+0.25\left[{\left(\delta_{p}^{API}-{\bar{\delta }}_{p}\right)}^{2}+{\left(\delta_{h}^{API}-{\bar{\delta }}_{h}\right)}^{2}\right]\right\}+\ln\frac{V_{API}}{\bar{V}}+1-\frac{V_{API}}{\bar{V}},$$

where V is the molar volume of ingredient.

The molar volume weighted Hansen solubility parameter ($\bar{\delta }$) can be calculated for each type of interaction (${\bar{\delta }}_{d},\;{\bar{\delta }}_{p},\;{\bar{\delta }}_{h}$) and for each ingredient of the mixture (k=drug substance or polymer) using Equation (4). In this equation *ϕ* is the volume fraction of drug substance or polymer (*k*) that can be calculated based on the mole fraction (x) (Equations (5) and (6)):

(4) $$\bar{\delta }=\sum_{k=1}^{n}\phi_{k}\delta_{k},$$

(5) $$\phi_{k}=\frac{x_{k}V_{k}}{\bar{V}},$$

(6) $$\bar{V}=\sum_{k=1}^{n}x_{k}V_{k},$$

(7) $$V_{k}=\frac{M_{k}}{\rho_{k}},$$

where $\bar{V}$ is the volume of the mixture, M the molecular weight and ρ the density of ingredient.

The values calculated as quantity of CAF that can be dissolved at a given temperature and mixture composition are summarized in Table 4. The extremes of these values were used to construct the solubility curve. At ~20 °C a small amount of CAF (up to a maximum at 2%) can be dissolved in HPMC. Increasing the temperature up to the melting point increases the percentage of solutes to over 70%.

**Figure 3:**
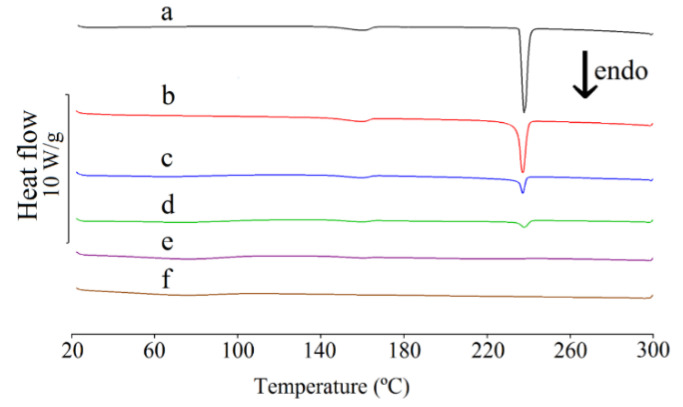
DSC curves for (a) caffeine and their mixtures containing: (b) 10%, (c) 30%, (d) 50%, (e) 70% and (f) 90% of HPMC.

The real effect of HPMC on the crystallinity of CAF was assessed by means of DSC studies. As shown in Figure 3 (curve a), a broad endothermic DSC peak in the temperature range of 140–160 °C can be assigned to the polymorphic transition of the anhydrous modification II of CAF to polymorphic form I [34]. This is consistent with CAF behavior during HSM tests. Microscopic observations showed that CAF recrystallized at ~145 °C to needle-shaped crystals due to the crystal interconversion of form II → form I. The melting point of CAF, reflected by a sharp endothermic DSC peak at ~237 °C, is also confirmed by HSM. In this particular study, the onset temperature of this peak was observed at 235 °C. These values are consistent with the literature data.

The DSC curves of CAF mixtures with HPMC are shown in Figure 3 (curves b–f). A broad peak in the temperature range of 140–160 °C due to the CAF polymorphic transitions was found on the DSC curves for all mixtures. This crystal interconversion commenced at ~145 °C, as additionally confirmed by the HSM measurements. The onset temperature of melting of CAF in the mixtures containing 10–50% of HPMC is consistent with CAF melting point. However, the melting point of CAF in the mixture containing 70% of HPMC is shifted to a lower value. Additionally, no DSC peak due to the melting of CAF was observed for the mixture containing 90% of HPMC, indicating that CAF does not exist in the crystalline form in this mixture. Again, these findings were confirmed by HSM. The onset temperature of the melting peak of modification I was observed above 195 °C for mixtures containing 10%, 30% and 50% of HPMC and at 188 °C for the mixture containing 70% of polymer. DSC peak due to the melting of CAF in the mixture containing 90% of HPMC was not found, instead only the liquefaction of the entire sample was observed at ~265 °C.

The DSC data listed in Table 5 confirmed that the heat of melting increases proportionally to the content of CAF in the mixtures with HPMC. This is consistent with the literature data [35,36,37]. The strong relationship between the heat of melting and the CAF content in a mixture (correlation coefficient r = 0.9661) can be described by the following equation:

(8) $$∆H_{f}=1.4287\;m-48.279$$

where Δ*H_f_* is the heat of fusion and *m* is the content of CAF in the mixture.

However, the straight line that reflected this relationship intersects the abscissa at ~33.8% of CAF content. The high and negative intercept value (−48.279) in the regression equation indicates a proportional reduction in CAF crystallinity, which confirms that CAF partially loses its crystallinity after homogenization with HPMC.

**Figure 4:**
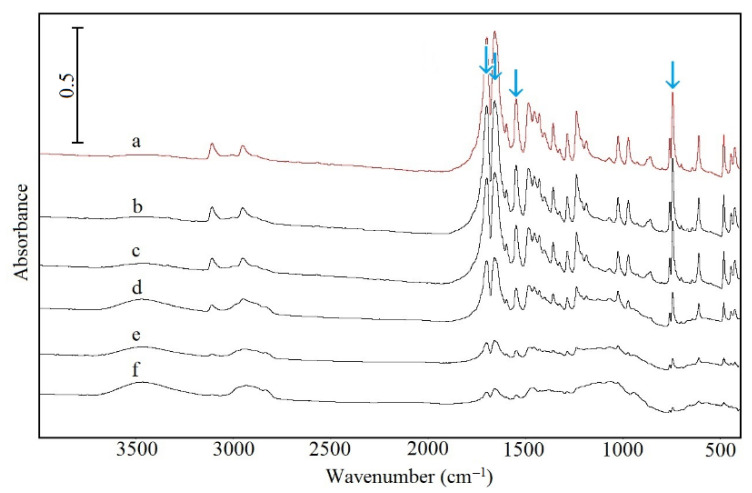
FTIR spectra for (a) caffeine and their mixtures containing: (b) 10%, (c) 30%, (d) 50%, (e) 70% and (f) 90% of HPMC.

**Figure 5:**
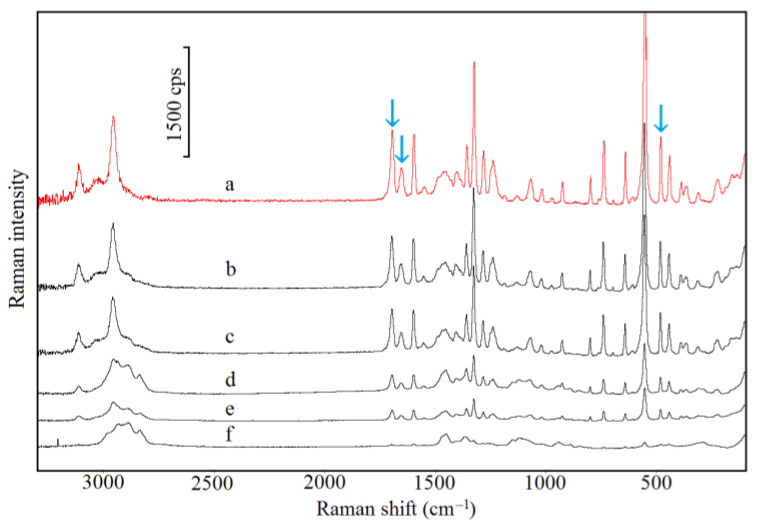
Raman spectra for (a) caffeine and their mixtures containing: (b) 10%, (c) 30%, (d) 50%, (e) 70% and (f) 90% of HPMC.

The FTIR and Raman spectra of CAF are shown in Figure 4 (curve a) and Figure 5 (curve a), respectively, whereas characteristic absorption bands and Raman shifts are compiled in Table 6. As shown in Figure 4 (curves b–f) and Figure 5 (curves b–f), the FTIR and Raman spectra for CAF mixtures with HPMC displayed all the bands characteristic of CAF. However, the intensity of these bands decreases in line with decreasing CAF content in the mixture. The bands that may reflect the formation of hydrogen bonds are marked with arrows (acceptor of free electron pair) [38]. Additionally, there are no new bands or significant changes in position as compared with the CAF spectrum.

To improve the interpretation of FTIR spectra, principal component analysis (PCA) was used. This is an advanced multivariate statistical technique based on the dimensionality reduction of huge sets of data, which increases the interpretability of such datasets with minimal loss of information. Two FTIR spectral ranges containing data on deformation vibrations of the O=C–C group of CAF (670–800 cm^–1^), asymmetric and symmetric stretching vibrations of the same group and stretching vibrations of the C=N group (1500–1800 cm^–1^) were used for PCA calculations. Stretching vibrations of the methyl groups of HPMC were observed at 2900 cm^–1^ [39]. Wide and atypical absorption bands precluded their use in the PCA calculations.

**Figure 6:**
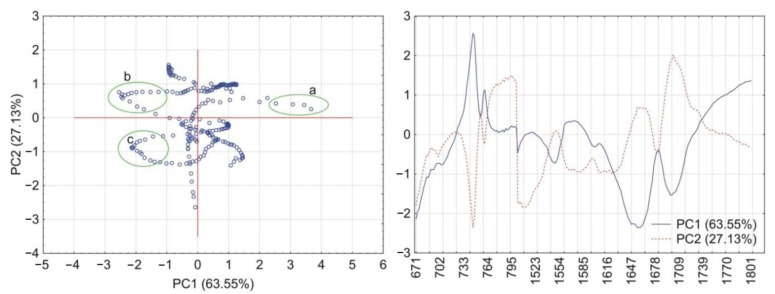
PCA plots of the data acquired from FTIR spectra of CAF and HPMC: scores scatter plot of PC1 and PC2 and corresponding loadings profiles of PC1 and PC2. Groups of scores highlights by circles indicate maximum wavenumbers of peaks displayed by loadings profiles at (a) 745 cm^–1^, (b) 1700 cm^–1^ and 1659 cm^–1^ (c).

**Figure 7:**
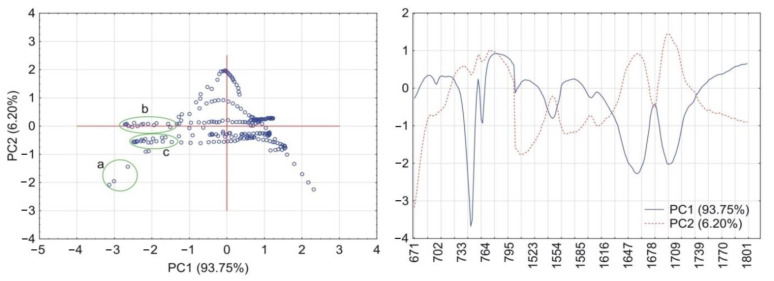
PCA plots of the data acquired from FTIR spectra of CAF mixtures with HPMC: scores scatter plot of PC1 and PC2 and corresponding loadings profiles of PC1 and PC2. Groups of scores highlights by circles indicate maximum wavenumbers of peaks displayed by loadings profiles at (a) 745 cm^–1^, (b) 1700 cm^–1^ and 1659 cm^–1^ (c).

PCA calculations for the matrix containing CAF and HPMC revealed that together PC1 and PC2 explained more than 90% of total variability. As illustrated in Figure 6, the PC1 loadings profile shows a positive value at the absorption band characteristic for vibrations of the O=C–C group (745 cm^–1^) and two negative values characteristic for vibrations of the C=O and C=N groups at 1659 cm^–1^ and 1700 cm^–1^, respectively. Hence, the wavenumber values for these groups were circled as groups of scores in the PC1 and PC2 plot. In the case of the matrix containing CAF mixtures with HPMC, the first two PCs explained almost 100% of total variability. Compared with the previous matrix, the PC1 loadings profile reveals one significant change (Figure 7) in that the absorption band characteristic to the O=C–C group shows a negative value. This change implies that the O=C–C group may participate in the formation of a hydrogen bond between CAF and HPMC in their mixtures.

### 3.2. Theophylline Mixtures with HPMC

**Figure 8:**
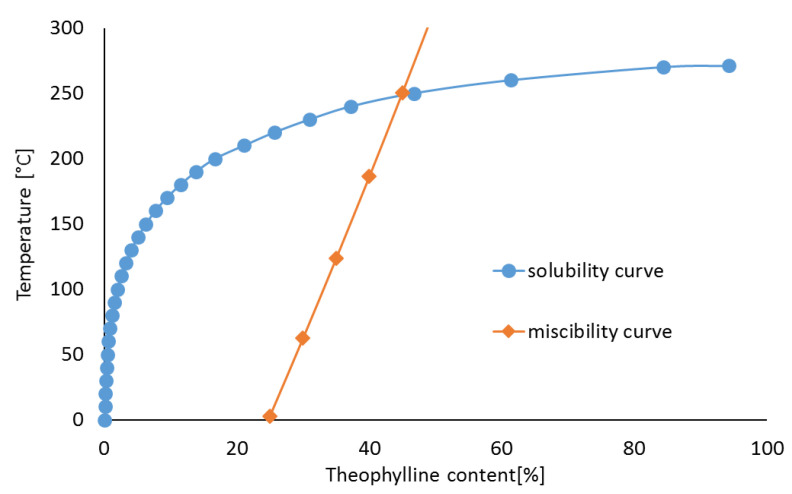
Phase diagram for theophylline binary mixtures with HPMC.

To assess the quantity of TF that may lose crystallinity in mixtures with amorphous HPMC, a phase diagram was developed. The miscibility and solubility curves calculated using Equations (1)–(7) are illustrated in Figure 8. The temperatures at which TF and HPMC are completely miscible at a given composition are listed in Table 3, while Table 7 shows the quantities of TF which may be dissolved in HPMC at a given temperature and composition. Calculations revealed that no more than 0.5% of TF can be dissolved in HPMC at 20 °C, but at TF melting point this proportion exceeds 90%.

**Figure 9:**
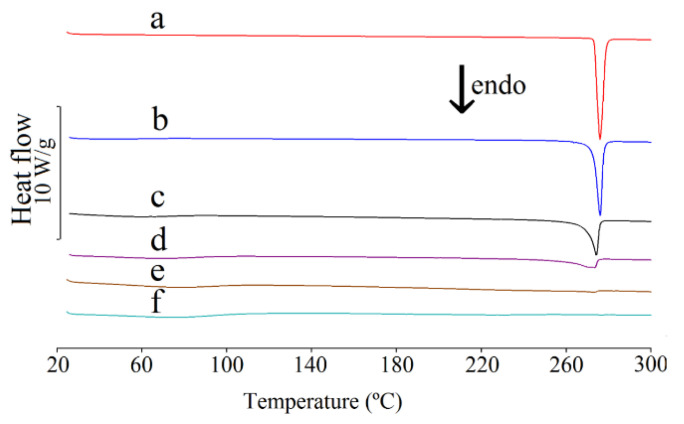
DSC curves for (a) theophylline and their mixtures containing: (b) 10%, (c) 30%, (d) 50%, (e) 70% and (f) 90% of HPMC.

The DSC curves of TF and its mixtures with HPMC are shown in Figure 9, while the temperatures and heats due to the melting of TF alone and its mixtures are summarized in Table 5. The onset of the melting peak for TF at ~272 °C is consistent with the literature data [40]. For the mixtures with HPMC, these temperatures are shifted to lower values. This is attributable to a decrease of content of methylxanthine in crystalline form in the mixtures. The relationship between the heat of melting of TF and its content in the mixture is linear and can be described by the equation:

(9) $$∆H_{f}=1.6796\;m-27.665$$

where Δ*H_f_* is the heat of fusion and *m* is the content of TF in the mixture.

This relationship is characterized by a high value correlation coefficient of r = 0.9930. However, the high value of the slope confirms that TF partially loses crystallinity after homogenization with HPMC, findings corroborated by HSM. The HSM measurements revealed that above 170 °C, slight sublimation begins for crystals of TF with crystallization of vapors on a coverslip in the form of needle-shaped crystals. Finally, TF melts at 270 °C. No other crystals or amorphous forms of TF and HPMC were formed as the mixtures were heated.

**Figure 10:**
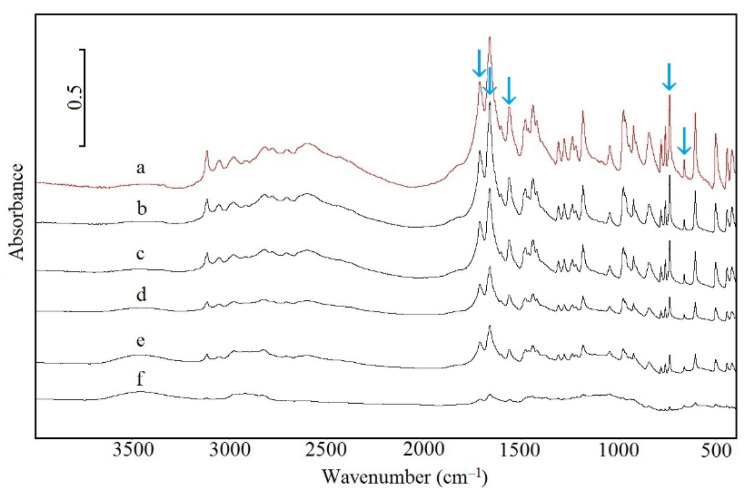
FTIR spectra for (a) theophylline and their mixtures containing: (b) 10%, (c) 30%, (d) 50%, (e) 70% and (f) 90% of HPMC.

**Figure 11:**
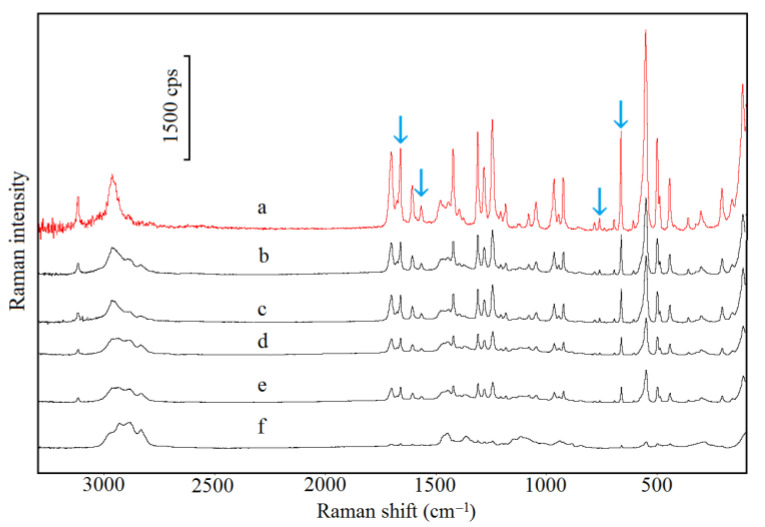
Raman spectra for (a) theophylline and their mixtures containing: (b) 10%, (c) 30%, (d) 50%, (e) 70% and (f) 90% of HPMC.

The characteristic FTIR absorption bands and Raman shifts for TF (Table 6; Figure 10, curve a; Figure 11, curve a) are consistent with the literature data [41]. The FTIR and Raman spectra of physical mixtures of TF with HPMC are shown in Figure 10 (curves b–f) and Figure 11 (curves b–f), respectively. These spectra verified that all the bands attributed to the chemical structure of methylxanthine were found in the spectra of its mixtures with HPMC. No new peaks in these spectra nor difference in position were found in comparison to the TF spectrum. Particular attention was paid to bands that would indicate hydrogen bond formation (acceptor of free electron pair). These bands are indicated by arrows.

**Figure 12:**
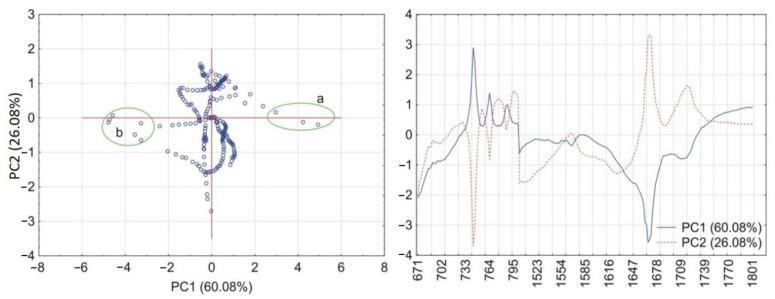
PCA plots of the data acquired from FTIR spectra of TF and HPMC: scores scatter plot of PC1 and PC2 and corresponding loadings profiles of PC1 and PC2. Groups of scores highlights by circles indicate maximum wavenumbers of peaks displayed by loadings profiles at (a) 742 cm^−1^ and (b) 1667 cm^−1^.

**Figure 13:**
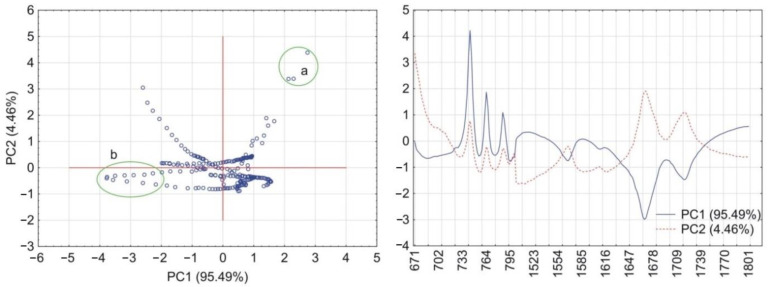
PCA plots of the data acquired from FTIR spectra of TF mixtures with HPMC: scores scatter plot of PC1 and PC2 and corresponding loadings profiles of PC1 and PC2. Groups of scores highlights by circles indicate maximum wavenumbers of peaks displayed by loadings profiles at (a) 742 cm^−1^ and (b) 1667 cm^−1^.

The results of PCA calculations for the acquired data from the FTIR spectra are shown in Figure 12 (matrix with TF and HPMC) and Figure 13 (TF mixtures with HPMC). For both matrices, the PC1 and PC2 explained, respectively, more than 86% and almost 100% of total variability. The PC1 loadings profile for the matrix containing TF and HPMC (Figure 12) shows a positive value at the absorption band assigned to vibrations of the O=C–C group (742 cm^–1^) and a negative value attributed to asymmetric stretching vibrations of the C=O group at 1667 cm^–1^. The values of wavenumbers indicated by the loadings profile were circled in the PC1 and PC2 scores scatter plot. The PC1 loadings for the matrix containing TF mixtures with HPMC (Figure 13) reveals a similar profile to the previous matrix. However, a much higher variability at the PC1 positive value of the absorption band of the O=C–C group (742 cm^–1^) may imply a role in the formation of a hydrogen bond in the mixtures of TF with HPMC. The PC1 and PC2 loadings of absorption band at 1718 cm^–1^ assigned to stretching vibrations of the C=N group did not differ in the case of either matrix. Therefore, the participation of this functional group in the formation of hydrogen bonds can most likely be excluded.

## 4. Discussion

The findings of this work revealed that three areas can be separated in the phase diagram. On the left-hand side of the solubility curve, the solid solution created by crystalline methylxanthine and amorphous polymeric excipient is stable; on the right-hand side of the miscibility curve, both ingredients show a tendency towards separation. In the area between these curves the solid solution is thermodynamically unstable. Thus, taking into account the miscibility of ingredients, the phase diagrams show the quantities of CAF and TF which can create stable systems with HPMC by the formation of solid solution. The formation of solid solutions and amorphization were also confirmed using the phase diagrams created for felodipine and nitrendipine mixtures with polyvinylpyrrolidone [16].

DSC measurements confirmed that some of the CAF and TF in the binary physical mixtures with HPMC lose their crystallinity. Based on the DSC peaks due to the melting of methylxanthines, linear relationships were found between the heats of melting of CAF and TF and their contents in binary mixtures with HPMC. However, these linear relationships show high negative intercept values. This indicates a proportional loss of crystallinity of CAF and TF due to the formation of solid solutions. In this way, CAF and TF in mixtures with polymeric excipient partially undergo amorphization, a process also observed in the TF mixtures with methylcellulose and chitosan [22,23]. Moreover, HSM measurements reveal that no other crystalline or amorphous forms other than those observed for CAF and TF were created as the mixtures underwent heating.

The chemical structure of methylxanthines shows an oxygen atom as a part of two carbonyl groups and additionally, a nitrogen atom as a part of the imidazole ring [38,41,42]. Both the oxygen and nitrogen atoms can play the role of acceptors of free electron pairs as hydrogen bonds are formed, allowing the CAF and TF to create hydrogen bonding. Additionally, it is noteworthy that for HPMC the methyl groups can participate in the formation of hydrogen bonds as donors of free electron pairs [43]. An analysis of the position and intensity of the bands in the spectra reveals the presence of all the peaks characteristic for methylxanthines.

When employed as a tool to support interpretation of the FTIR spectra of methylxanthines and their mixtures with polymeric excipient, PCA permits the identification of those spectral bands characterized by the greatest variability. The loadings profiles of PC1 and PC2 reveal that bands exist which correspond to the chemical groups involved in the creation of hydrogen interactions between ingredients. The reduction of methylxanthine crystallinity may therefore proceed with the formation of hydrogen interactions.

## 5. Conclusions

The measurements performed reveal that the miscibility of ingredients and formation of solid solution are effective in the partial reduction of methylxanthine crystallinity when mixed with HPMC. This is described mathematically by the phase diagrams, which graphically show the conditions at which thermodynamically distinct phases exist or coexist at equilibrium. The diagram developed also allows an evaluation of the proportions at which homogenization of methylxanthines with HPMC will create thermodynamically stable solid solutions. Therefore, amorphization of CAF and TF by homogenization with HPMC can be applied in the pharmaceutical technology to manufacture the solid dosage formulations, which may contain lower doses of both methylxanthines in pharmaceutical product due to better solubility and dissolution rate of amorphous drug substance. Additionally, DSC was found to be the most efficient tool to confirm the partial reduction of CAF and TF crystallinity. On the other hand, the PCA technique enables the identification of the functional groups responsible for the formation of hydrogen bonding.

## Figures and Tables

**Table 1:** Bioavailability parameters of an active substance in crystalline or amorphous form.

Drug Substance	Polymorphic/Amorphous Form	*AUC* (μg·h/mL)	*C_max_* (μg/mL)	*T_max_* (h)	Ref.
Itraconazole	crystalline		10.5 ± 8.6 **	6.0 ± 2.0	5
	amorphous (SD with HPMC-P)		340.7 ± 27.2 **	1.7 ± 0.5	
Atorvastatin calcium	Lipitor (commercial product)	534.5 ± 278.3 ^(1)^ *	338.7 ± 80.4 **	0.7 ± 0.2	6
	amorphous (SD with Poloxamer 188)	919.0 ± 315.1 ^(1)^ *	972.2 ± 174.5 **	0.6 ± 0.2	
Oleanolic acid	commercial tablet	761.8 ± 272.2 ^(2)^ *	89.1 ± 33.1 **	1.8 ± 1.6	7
	amorphous (SD with PVP)	1840 ± 381.8 ^(2)^ *	498.7 ± 120.8 **	1.1 ± 0.5	
	crystalline (PM with PVP)	615.1 ± 115.5 ^(2)^ *	134.4 ± 47.4 **	1.1 ± 1.0	
Apigenin	crystalline	146.5 ± 62.4 ^(2)^	21.4 ± 6.4	3.6 ± 0.8	8
	commercial capsule	142.0 ± 30.1 ^(2)^	17.5 ± 1.3	4.0 ± 0.7	
	amorphous (SD with Pluronic F-127, microwave method)	453.2 ± 328.8 ^(2)^	60.5 ± 24.7	2.8 ± 1.3	
	amorphous (SD with Pluronic F-127, melted method)	187.4 ± 83.4 ^(2)^	31.8 ± 21.2	2.8 ± 1.8	
	partially amorphous (SD with Pluronic F-127, kneaded method)	83.4 ± 19.4 ^(2)^	11.8 ± 4.4	3.2 ± 1.8	
Silymarin	crystalline	2.1 ± 0.1 ^(3)^	0.6 ± 0.1	1.5 ± 0.4	9
	amorphous (SD with PVP K17)	5.0 ± 0.4 ^(3)^	1.1 ± 0.2	0.5 ± 0.1	
Valsartan	crystalline	10.6 ± 0.8 ^(4)^	0.7 ± 0.1	1.0 ± 0.2	10
	commercial product	13.66 ± 2.9 ^(4)^	0.8 ± 0.1	1.2 ± 0.1	
	amorphous (SD with HPMC and SLS)	23.6 ± 2.6 ^(4)^	2.5 ± 0.2	0.7 ± 0.1	
Ezetimibe	crystalline	3.1 ± 0.7	0.3 ± 0.3	1.4 ± 0.9	11
	amorphous (SD with HPC)	4.9 ± 0.5	0.5 ± 0.1	0.7 ± 0.3	
	amorphous (SD with HPC and Tween 80)	5.4 ± 0.2	0.9 ± 0.1	1.2 ± 0.3	

^(1)^ AUC_0–8_, ^(2)^ AUC_0–24_, ^(3)^ AUC_0–t_, ^(4)^ AUC_0–∞_, * [ng·h/mL], ** [ng/mL], HPC—hydroksypropyl cellulose, HPMC-P—hydroksypropyl methylcellulose phthalate, PVP—poly(vinyl pyrrolidone), SLS—sodium lauryl sulfate, SD—solid dispersion, PM—physical mixture.

**Table 2:** The parameters used to develop the phase diagrams.

Parameter	HPMC	Caffeine	Theophylline
Molar mass (g/mol)	10,000	194.19 [28]	180.16 [30]
Density (g/cm^3^)	1.0 [27]	1.23 [28]	1.35 [30]
Heat of melting (J/mol)	–	19,736	28,264
Molar volume (cm^3^/mol)	–	157.9 [28]	133.45
Volume of a lattice (Å^3^)	–	827.1 [29]	898.43 [31]
Glass transition (K)	433.15	-	-
Melting (K)	–	509.34	544.97
**Solubility Parameters (MPa)^1/2^**			
δ_d_	16.95 [27]	19.5 [28]	17.0 [30]
δ_p_	8.55 [27]	10.1 [28]	11.0 [30]
δ_h_	9.04 [27]	13.0 [28]	12.0 [30]
δ	21.03	25.52	23.53

**Table 3:** The temperatures of miscibility of methylxanthines in HPMC.

Content of Drug Substance (%)	Mole Fraction (*x*)		Volume Fraction (*ɸ*)		Temperature of Miscibility (°C)
	**Caffeine**	**HPMC**	**Caffeine**	**HPMC**	
9	0.8674	0.1326	0.073	0.927	25.60
10	0.8802	0.1198	0.081	0.919	59.38
11	0.8910	0.1090	0.089	0.911	93.27
12	0.9002	0.0998	0.098	0.902	127.28
13	0.9081	0.0919	0.106	0.894	161.40
14	0.9150	0.0850	0.114	0.886	195.65
15	0.9211	0.0789	0.123	0.877	230.01
	**Theophylline**	**HPMC**	**Theophylline**	**HPMC**	
25	0.9566	0.0434	0.209	0.791	2.87
30	0.9659	0.0341	0.254	0.746	62.41
35	0.9727	0.0273	0.299	0.701	123.49
40	0.9778	0.0222	0.346	0.654	186.13
45	0.9819	0.0181	0.394	0.606	250.35

**Table 4:** The quantities of caffeine which can be dissolved in HPMC at a given temperature.

Temperature (°C)	0	10	20	30	40	50	60	70	80	90	100	110	120	130	140	150	160	170	180	190	200	210	220	230
Caffeine (%)	Caffeine Dissolved in HPMC at a Given Temperature (%)																							
2	0.44	0.61	0.83	1.10	1.44	1.86																		
3	0.44	0.62	0.84	1.12	1.46	1.88	2.38	2.98																
4	0.45	0.63	0.85	1.13	1.48	1.91	2.41	3.02	3.72															
5	0.46	0.64	0.86	1.15	1.50	1.93	2.45	3.05	3.77	4.59														
6	0.46	0.64	0.88	1.17	1.52	1.96	2.48	3.09	3.81	4.65	5.61													
7	0.47	0.65	0.89	1.18	1.54	1.98	2.51	3.13	3.86	4.71	5.68	6.78												
8	0.48	0.66	0.90	1.20	1.56	2.01	2.54	3.17	3.91	4.76	5.74	6.86												
9	0.48	0.67	0.91	1.21	1.58	2.03	2.57	3.21	3.96	4.82	5.81	6.94	8.21											
10	0.49	0.68	0.92	1.23	1.60	2.06	2.61	3.25	4.01	4.88	5.88	7.02	8.31											
11	0.50	0.69	0.94	1.25	1.63	2.09	2.64	3.29	4.06	4.94	5.95	7.11	8.41											
12	0.51	0.70	0.95	1.26	1.65	2.11	2.67	3.33	4.11	5.00	6.03	7.19	8.50	9.97										
13	0.51	0.71	0.96	1.28	1.67	2.14	2.71	3.38	4.16	5.06	6.10	7.28	8.60	10.09	11.74									
14	0.52	0.72	0.98	1.30	1.69	2.17	2.74	3.42	4.21	5.12	6.17	7.36	8.70	10.20	11.87									
15	0.53	0.73	0.99	1.31	1.71	2.20	2.77	3.46	4.26	5.18	6.24	7.45	8.80	10.32	12.01	13.87								
16	0.53	0.74	1.00	1.33	1.73	2.22	2.81	3.50	4.31	5.25	6.32	7.53	8.91	10.44	12.14	14.02								
17	0.54	0.75	1.01	1.35	1.76	2.25	2.84	3.54	4.36	5.31	6.39	7.62	9.01	10.56	12.28	14.18								
18	0.55	0.76	1.03	1.36	1.78	2.28	2.88	3.59	4.42	5.37	6.47	7.71	9.11	10.68	12.42	14.34	16.45							
19	0.56	0.77	1.04	1.38	1.80	2.31	2.91	3.63	4.47	5.44	6.54	7.80	9.22	10.80	12.56	14.50	16.63							
20	0.56	0.78	1.05	1.40	1.82	2.34	2.95	3.68	4.52	5.50	6.62	7.89	9.32	10.92	12.70	14.66	16.81	19.16						
25	0.60	0.83	1.12	1.49	1.94	2.48	3.13	3.90	4.79	5.83	7.01	8.35	9.86	11.54	13.41	15.48	17.74	20.21	22.89					
30	0.64	0.89	1.20	1.58	2.06	2.63	3.32	4.13	5.07	6.16	7.41	8.82	10.41	12.18	14.15	16.32	18.70	21.30	24.11					
35	0.68	0.94	1.27	1.68	2.18	2.79	3.51	4.37	5.36	6.51	7.82	9.31	10.98	12.85	14.92	17.20	19.69	22.42	25.38	28.57				
40	0.72	1.00	1.34	1.78	2.31	2.95	3.71	4.61	5.66	6.87	8.25	9.81	11.57	13.53	15.70	18.09	20.71	23.57	26.67	30.02	33.62			
45	0.76	1.05	1.42	1.88	2.43	3.11	3.91	4.86	5.96	7.23	8.68	10.32	12.16	14.22	16.50	19.01	21.76	24.75	28.00	31.51	35.27	39.31		
50	0.81	1.11	1.50	1.97	2.56	3.27	4.12	5.11	6.26	7.60	9.12	10.84	12.77	14.92	17.31	19.94	22.82	25.95	29.35	33.02	36.97	41.19		
55	0.85	1.17	1.57	2.07	2.69	3.43	4.32	5.36	6.57	7.96	9.56	11.36	13.38	15.64	18.13	20.88	23.89	27.17	30.73	34.56	38.68	43.10		
60	0.89	1.22	1.65	2.17	2.82	3.59	4.52	5.61	6.88	8.33	10.00	11.88	13.99	16.35	18.96	21.83	24.98	28.40	32.11	36.12	40.42	45.03	49.94	
65	0.93	1.28	1.72	2.27	2.94	3.75	4.72	5.86	7.18	8.70	10.43	12.40	14.60	17.06	19.78	22.78	26.06	29.63	33.50	37.68	42.17	46.97	52.10	57.54
70	0.97	1.33	1.79	2.36	3.06	3.91	4.91	6.09	7.47	9.05	10.86	12.91	15.20	17.76	20.60	23.72	27.14	30.86	34.89	39.24	43.92	48.92	54.25	59.92
75	1.00	1.38	1.85	2.45	3.18	4.05	5.10	6.33	7.75	9.40	11.28	13.40	15.79	18.45	21.40	24.64	28.19	32.06	36.26	40.78	45.65	50.85	56.40	62.30
80	1.03	1.42	1.92	2.53	3.28	4.19	5.27	6.54	8.02	9.73	11.68	13.88	16.35	19.11	22.17	25.54	29.23	33.24	37.60	42.30	47.35	52.75	58.52	64.65
85	1.06	1.46	1.97	2.60	3.38	4.31	5.43	6.74	8.27	10.04	12.05	14.33	16.89	19.74	22.91	26.40	30.22	34.38	38.89	43.76	49.00	54.61	60.59	66.95
90	1.08	1.49	2.02	2.67	3.46	4.43	5.57	6.93	8.50	10.32	12.39	14.74	17.39	20.33	23.60	27.21	31.16	35.46	40.13	45.17	50.59	56.39	62.59	69.17
95	1.10	1.52	2.05	2.72	3.53	4.52	5.70	7.08	8.70	10.57	12.70	15.12	17.84	20.87	24.24	27.96	32.03	36.47	38.43	46.49	52.09	58.09	64.49	71.31

**Table 5:** The heats of fusion and the onset and peak temperatures of the melting of methylxanthines in the mixtures in HPMC.

**Content of Caffeine in Mixture (%)**	**Heat of Fusion (J/g)** **Δ*H_f_***	**Onset Temperature (°C)** ***T_on_***	**Peak Temperature (°C)** ***T_p_***
10	–	–	–
30	3.7	220.6	233.5
50	20.5	235.3	239.2
70	34.2	235.9	238.3
90	82.5	235.5	237.3
100	103.5	236.9	237.9
**Content of Theophylline in Mixture (%)**	**Heat of Fusion (J/g)** **Δ** ***H_f_***	**Onset Temperature (°C)** ***T_on_***	**Peak Temperature (°C)** ***T_p_***
10	–	–	–
30	20.9	249.5	268.0
50	62.1	261.8	270.2
70	83.8	268.4	272.3
90	125.7	271.0	273.4
100	155.3	271.9	273.1

**Table 6:** The characteristic bands of methylxanthines in FTIR and Raman spectra.

FTIR (cm^–1^)			Raman (cm^–1^)		
This Study	[38]	Assignment	This Study	[38]	Assignment
**Caffeine**					
481	481	δ *a* C–N–C	226	225	δ N–C–N
610	611	δ C=C–C	442	444	δ N–C–C
745	743	δ O=C–C	481	488	δ *a* C–N–C
860	862	δ N=C–H	553	556	δ C-N–CH_3_
973	973	ν N–CH_3_	641	645	δ C=C–N
1025	1025	ν *a* N–CH_3_	739	745	δ C=C–C
1188	1189	ν CC, ν *a* CN	799	800	δ N–C–H
1239	1237	ν CN	925	925	ν *s* N–CH_3_
1286	1285	ν CN	1019	1020	ν *a* N–CH_3_
1549	1548	ν *s* C=O	1240	1241	ν CN
1598	1600	ν C=C	1282	1288	ν CN
1659	1660	ν *a* C=O	1326	1331	ν CN
1699	1700	ν C=N	1598	1600	ν C=C
2953	2954	ν CH	1654	1656	ν *a* C=O
			1696	1700	ν C=N
			2958	2963	ν CH
**Theophylline**					
446	450	δ N–C–C	444	450	δ *a* C–N–C
504	500	δ *a* C–N–C	552	550	δ C=C–C
610	615	δ C=C–N	608	610	δ C=C–N
667	670	δ O=C–N	664	660	δ O=C–N
742	744	δ O=C–C	762	760	δ O=C–C
848	850	δ C–N–H	925	920	δ N–C–N
927	925	ν *s* N–CH_3_	947	940	ν *s* N–CH_3_
979	980	ν *a* N–CH_3_	967	970	δ N=C–H
1241	1240	ν CN	1082	1088	ν *s* C–N
1284	1285	ν CN	1246	1244	ν C–N
1313	1310	ν CN	1284	1280	ν C–N
1567	1556	ν *s* C=O	1568	1570	ν *s* C=O
1667	1664	ν *a* C=O	1609	1613	ν C=C
1718	1710	ν C=N	1662	1663	ν *a* C=O
			1704	1706	ν C=N
			3121	3120	ν N–H

Vibrations: ν—stretching, δ—deformation, *a*—asymmetric, *s*—symmetric.

**Table 7:** The quantities of theophylline which can be dissolved in HPMC at a given temperature.

Temperature (°C)	0	10	20	30	40	50	60	70	80	90	100	110	120	130	140	150	160	170	180	190	200	210	220	230	240	250	260	270
Theophylline (%)	Theophylline Dissolved in HPMC at a Given Temperature (%)																											
2	0.06	0.09	0.15	0.22	0.32	0.45	0.62	0.85	1.14	1.50	1.95																	
3	0.06	0.10	0.15	0.22	0.32	0.45	0.63	0.86	1.15	1.51	1.97	2.52																
4	0.06	0.10	0.15	0.22	0.32	0.46	0.64	0.87	1.16	1.53	1.99	2.55	3.22															
5	0.06	0.10	0.15	0.22	0.33	0.46	0.64	0.88	1.17	1.55	2.01	2.57	3.26	4.07														
6	0.06	0.10	0.15	0.23	0.33	0.47	0.65	0.89	1.19	1.56	2.03	2.60	3.29	4.11	5.08													
7	0.06	0.10	0.15	0.23	0.33	0.47	0.66	0.90	1.20	1.58	2.05	2.63	3.32	4.15	5.14	6.29												
8	0.06	0.10	0.16	0.23	0.34	0.48	0.67	0.91	1.21	1.60	2.08	2.66	3.36	4.20	5.19	6.35	7.70											
9	0.07	0.10	0.16	0.24	0.34	0.49	0.67	0.92	1.23	1.62	2.10	2.69	3.39	4.24	5.24	6.42	7.78											
10	0.07	0.11	0.16	0.24	0.35	0.49	0.68	0.93	1.24	1.63	2.12	2.71	3.43	4.29	5.30	6.48	7.86	9.44										
11	0.07	0.11	0.16	0.24	0.35	0.50	0.69	0.94	1.26	1.65	2.14	2.74	3.47	4.33	5.35	6.55	7.94	9.54										
12	0.07	0.11	0.16	0.24	0.36	0.50	0.70	0.95	1.27	1.67	2.17	2.77	3.50	4.38	5.41	6.61	8.02	9.63	11.48									
13	0.07	0.11	0.17	0.25	0.36	0.51	0.71	0.96	1.28	1.69	2.19	2.80	3.54	4.42	5.46	6.68	8.10	9.73	11.59									
14	0.07	0.11	0.17	0.25	0.36	0.51	0.71	0.97	1.30	1.71	2.22	2.83	3.58	4.47	5.52	6.75	8.18	9.83	11.71	13.84								
15	0.07	0.11	0.17	0.25	0.37	0.52	0.72	0.98	1.31	1.73	2.24	2.86	3.62	4.52	5.58	6.82	8.26	9.92	11.82	13.98								
16	0.07	0.11	0.17	0.26	0.37	0.53	0.73	0.99	1.33	1.75	2.26	2.89	3.66	4.56	5.64	6.89	8.35	10.02	11.94	14.12								
17	0.07	0.11	0.18	0.26	0.38	0.53	0.74	1.01	1.34	1.77	2.29	2.93	3.69	4.61	5.69	6.96	8.43	10.13	12.06	14.26	16.74							
18	0.07	0.12	0.18	0.26	0.38	0.54	0.75	1.02	1.36	1.79	2.31	2.96	3.73	4.66	5.75	7.03	8.52	10.23	12.18	14.40	16.90							
19	0.07	0.12	0.18	0.27	0.39	0.55	0.76	1.03	1.37	1.81	2.34	2.99	3.77	4.71	5.81	7.11	8.61	10.33	12.30	14.54	17.07							
20	0.08	0.12	0.18	0.27	0.39	0.55	0.77	1.04	1.39	1.83	2.37	3.02	3.82	4.76	5.88	7.18	8.69	10.44	12.43	14.69	17.23							
25	0.08	0.13	0.19	0.29	0.42	0.59	0.81	1.10	1.47	1.93	2.50	3.19	4.03	5.02	6.19	7.56	9.15	10.98	13.07	15.44	18.10	21.10						
30	0.09	0.14	0.21	0.31	0.44	0.62	0.86	1.17	1.56	2.04	2.64	3.37	4.25	5.29	6.53	7.97	9.64	11.55	13.74	16.23	19.03	22.16	25.65					
35	0.09	0.14	0.22	0.32	0.47	0.66	0.91	1.24	1.65	2.16	2.79	3.56	4.49	5.59	6.88	8.40	10.15	12.16	14.46	17.07	20.00	23.29	26.95	31.00				
40	0.10	0.15	0.23	0.35	0.50	0.70	0.97	1.31	1.74	2.29	2.95	3.76	4.74	5.90	7.26	8.85	10.69	12.81	15.23	17.96	21.04	24.48	28.32	32.56	37.24			
45	0.11	0.16	0.25	0.37	0.53	0.74	1.03	1.39	1.85	2.42	3.12	3.98	5.00	6.22	7.66	9.33	11.27	13.50	16.03	18.90	22.13	25.75	29.77	34.22	39.12			
50	0.11	0.17	0.27	0.39	0.56	0.79	1.09	1.47	1.96	2.56	3.30	4.20	5.28	6.57	8.08	9.84	11.88	14.22	16.88	19.90	23.29	27.09	31.30	35.97	41.11	46.75		
55	0.12	0.19	0.28	0.42	0.60	0.84	1.15	1.56	2.07	2.71	3.49	4.44	5.58	6.94	8.53	10.38	12.53	14.99	17.79	20.95	24.52	28.50	32.93	37.83	43.22	49.13		
60	0.13	0.20	0.30	0.44	0.63	0.89	1.22	1.65	2.19	2.87	3.69	4.69	5.90	7.32	9.00	10.95	13.21	15.79	18.74	22.07	25.81	29.99	34.64	39.78	45.44	51.64		
65	0.14	0.21	0.32	0.47	0.67	0.94	1.30	1.75	2.32	3.03	3.90	4.96	6.23	7.73	9.49	11.55	13.92	16.65	19.74	23.24	27.18	31.57	36.45	41.85	47.79	54.29	61.39	
70	0.14	0.22	0.34	0.50	0.71	1.00	1.37	1.85	2.45	3.20	4.12	5.24	6.57	8.16	10.02	12.18	14.68	17.54	20.80	24.48	28.61	33.23	38.36	44.03	50.26	57.09	64.54	
75	0.15	0.24	0.36	0.53	0.75	1.06	1.45	1.96	2.60	3.39	4.36	5.53	6.94	8.61	10.57	12.84	15.47	18.49	21.91	25.78	30.13	34.98	40.37	46.32	52.87	60.04	67.86	
80	0.16	0.25	0.38	0.56	0.80	1.12	1.54	2.07	2.74	3.58	4.60	5.84	7.32	9.08	11.14	13.54	16.30	19.47	23.08	27.15	31.72	36.82	42.48	48.73	55.61	63.14	71.35	
85	0.17	0.27	0.40	0.59	0.84	1.18	1.62	2.18	2.89	3.77	4.85	6.16	7.72	9.57	11.74	14.26	17.17	20.51	24.29	28.57	33.38	38.74	44.69	51.26	58.49	66.40	75.02	
90	0.18	0.28	0.42	0.62	0.89	1.25	1.71	2.30	3.05	3.98	5.11	6.49	8.13	10.08	12.36	15.01	18.08	21.58	25.57	30.06	35.11	40.75	47.00	53.91	61.50	69.81	78.87	88.70
95	0.19	0.30	0.45	0.66	0.94	1.31	1.80	2.43	3.21	4.19	5.38	6.83	8.55	10.60	13.00	15.79	19.01	22.70	20.81	31.61	36.92	42.84	49.41	56.67	64.65	73.38	82.89	93.22

## Data Availability

Data is contained within this article.
